# Rapid Identification of Common Secondary Metabolites of Medicinal Herbs Using High-Performance Liquid Chromatography with Evaporative Light Scattering Detector in Extracts

**DOI:** 10.3390/metabo11080489

**Published:** 2021-07-28

**Authors:** Kiran Ali, Arslan Ali, Muhammad Noman Khan, Saeedur Rahman, Shaheen Faizi, Muhammad Shaiq Ali, Shaden A. M. Khalifa, Hesham R. El-Seedi, Syed Ghulam Musharraf

**Affiliations:** 1 H.E.J. Research Institute of Chemistry, International Center for Chemical and Biological Sciences, Faculty of Science, University of Karachi, Karachi 75270, Pakistan; alikiran008@gmail.com (K.A.); Noman.muhammad@iccs.edu (M.N.K.); saeedsohail4@gmail.com (S.R.); Shaheen.faizi@iccs.edu (S.F.); shaiq.ali@iccs.edu (M.S.A.); 2 Dr. Panjwani Center for Molecular Medicine and Drug Research, International Center for Chemical and Biological Sciences, Faculty of Science, University of Karachi, Karachi 75270, Pakistan; arslanali1986@gmail.com; 3 Department of Molecular Biosciences, The Wenner-Gren Institute, Stockholm University, SE-106 91 Stockholm, Sweden; shaden.khalifa@su.se; 4 Department of Chemistry, Faculty of Science, Menoufia University, Shebin El-Kom 32512, Egypt; 5 International Research Center for Food Nutrition and Safety, Jiangsu University, Zhenjiang 212013, China; 6 T.C.M. Hospital of Southwest Medical University, Luzhou 646000, China

**Keywords:** dereplication, HPLC-ELSD, plant extracts, flavonoids, triterpenes, sterols

## Abstract

The discovery and identification of novel natural products of medicinal importance in the herbal medicine industry becomes a challenge. The complexity of this process can be reduced by dereplication strategies. The current study includes a method based on high-performance liquid chromatography (HPLC), using the evaporative light scattering detector (ELSD) to identify the 12 most common secondary metabolites in plant extracts. Twelve compounds including rutin, taxifolin, quercetin, apigenin, kaempferol, betulinic acid, oleanolic acid, betulin, lupeol, stigmasterol, and β-sitosterol were analyzed simultaneously. The polarity of the compounds varied greatly from highly polar (flavonoids) to non-polar (triterpenes and sterols). This method was also tested for HPLC-DAD and HPLC-ESI-MS/MS analysis. Oleanolic acid and ursolic acid could not be separated in HPLC-ELSD analysis but were differentiated using LC-ESI-MS/MS analysis due to different fragment ions. The regression values (R^2^ > 0.996) showed good linearity in the range of 50–1000 µg/mL for all compounds. The range of LOD and LOQ values were 7.76–38.30 µg/mL and 23.52–116.06 µg/mL, respectively. %RSD and % trueness values of inter and intraday studies were mostly <10%. This method was applied on 10 species of medicinal plants. The dereplication strategy has the potential to facilitate and shorten the identification process of common secondary metabolites in complex plant extracts.

## 1. Introduction

Herbal products have proven effective for the treatment of various diseases since ancient times and are still a major interest of researchers [1,2,3]. This is due to diverse bioactivities of the natural products and their potential as drug leads [1,4,5,6]. Many of the synthetic drugs have been designed by mimicking the unique structures of natural products, hence imparting their pharmaceutical properties [1,7,8]. Distinguishing between the already known compounds and the new unknown components in a plant mixture employing a screening analysis can be very useful. This is carried out using dereplication, a procedure that helps in the recognition of reported compounds at an early stage, thus making it easier to discriminate peaks of known compounds from the peaks of interest. This is an important step towards the discovery of novel compounds [2,9]. Dereplication strategies have been applied on several plant species to achieve different objectives such as to selectively isolate bioactive compounds [10,11,12,13,14,15,16].

Plant-derived secondary metabolites such as flavonoids, sterols, and triterpenes are ubiquitous in the plant kingdom. Triterpenes such as lupeol, betulinic acid, betulin, ursolic acid and oleanolic acid and sterols such as stigmasterol and β-sitosterol are found commonly in plants. Among flavonoids, quercetin, rutin, kaempferol, apigenin, and taxifolin (dihydroquercetin) have been reported as the most commonly present secondary metabolites in plants [17,18,19,20,21,22]. For the analysis of triterpenes and sterols, gas chromatography is commonly employed [23,24]. However, liquid chromatography-based analysis is also documented in the literature [17,25,26]. Since flavonoids are chromophore-bearing compounds, they can therefore be detected by ultraviolet (UV) radiation. However, triterpenes, especially pentacyclic triterpenes, lack chromophores, thus give a low-intensity response or cannot be observed under UV [27,28,29]. Evaporative light scattering detector (ELSD) possesses a non-discriminative ability for all sorts of compounds and it is cost-effective compared to the other universal detectors such as gas chromatography mass spectrometry (GC-MS) and liquid chromatography mass spectrometry (LC-MS).

In the present work, we have developed a high-performance liquid chromatography by means of the evaporative light scattering detector (HPLC-ELSD) method for the rapid identification of the most common plant-derived secondary metabolites of both polar and non-polar nature applying the same gradient. This dereplication strategy of the twelve plant secondary metabolites is not reported in the literature to the best of our knowledge. Our method is comparatively less time-consuming and it includes both polar flavonoids and non-polar triterpenes and sterols; in contrast, the other conventional techniques involve non-polar triterpenes and sterols that are mostly analyzed by using isocratic elution and the run times are usually very long—up to two hours [25,26,30].

## 2. Results and Discussion

### 2.1. HPLC-ELSD Optimization

**Figure 1:**
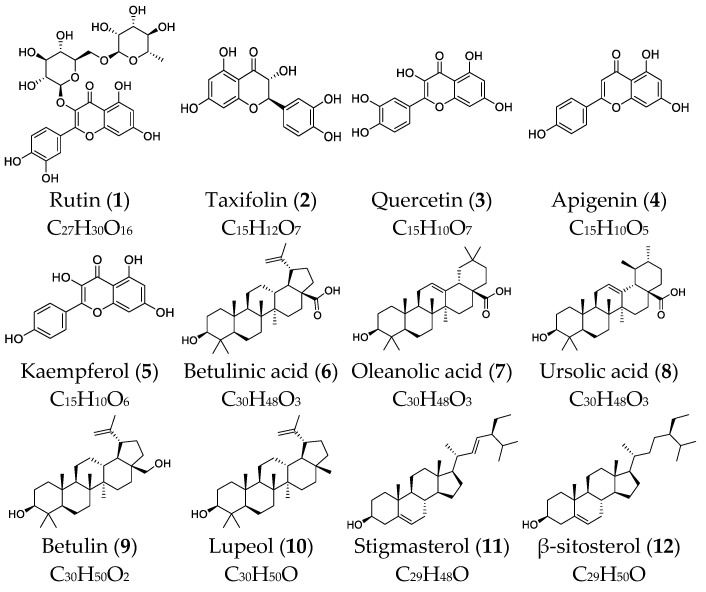
Secondary metabolites of plant origin used in the current study.

**Figure 2:**
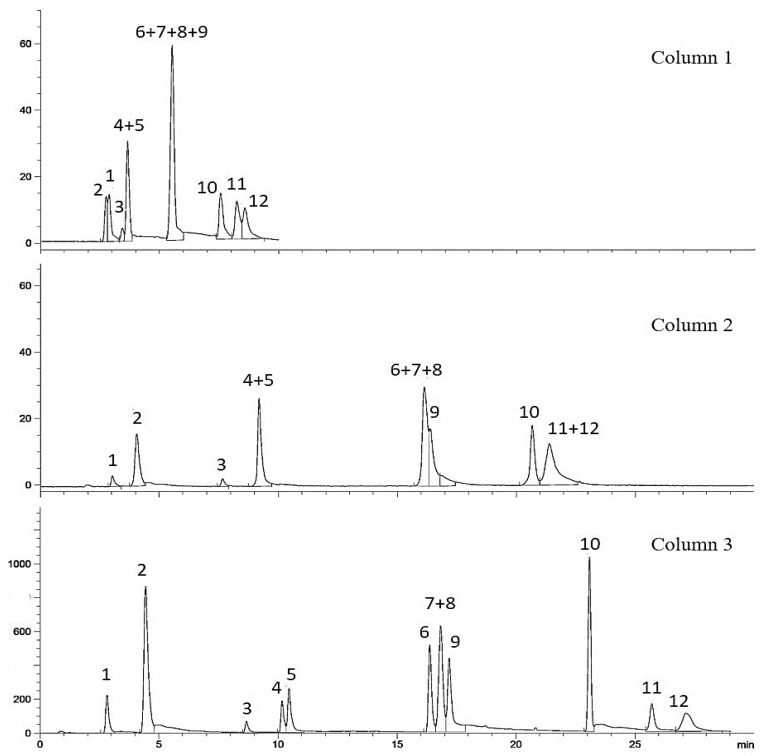
Comparison of chromatograms obtained on columns, **1**, **2**, and **3**.

A suitable stationary phase at a constant column temperature of 30 °C was chosen in the first step of method optimization. The standard compounds used in this step are given in Figure 1. Three different columns were tested, and column 1 displayed four different gradients for pool 1; gradient 4 was the best, in which eight peaks were observed. Rutin and taxifolin peaks were not fully resolved, as shown in Figure 2. The final chromatogram showed a clear peak shape and peak resolution. Nevertheless, three merged peaks were observed in correspondence to compounds **1**, **2**; **4**, **5**; and **6**–**9**. Compounds **6**, **7**, and **8** being isomeric posed the difficulty of their separation. The adopted solvent gradient is given in Table 1.

Similarly, four experiments were carried out using column 2. The best chromatogram, using gradient 4, showed eight peaks. The same peak interferences of compounds **4**, **5** and **6**–**9** were observed with an additional 11/12 merged peak (Figure 2). The peaks of compounds **1** and **2** were completely separated this time. Compared to column 1, peak shapes improved using column 2, but the total run time increased. The final gradient is shown in Table 1.

An injection of Pool-1 was also made on column 3 using seven different gradients. The best chromatogram obtained, using gradient 7, showed eleven peaks with retention times. Under the optimized conditions for this method (Table 1), compounds **7** and **8** remained unseparated, as shown in Figure 2. The peaks which were not completely resolved on column 1 and 2 appeared separately on column 3 and was enhanced visually. Peak resolution values of the seven gradients and the optimized gradient are presented in Supplementary Table S3 and Table 2, respectively.

The resolution for each peak was calculated using the formula Rs = 2 (tRB − tRA)/wA + wB. Rs shows resolution value, whereas tRA and tRB indicate the retention times of the peak under consideration (A) and the closest peak (B), respectively. wA + wB show peak widths of peak A and B, respectively, where the total run time was 29 min. The column used in this method was selected as the most appropriate stationary phase as well as the gradient used as the final gradient i.e., 7. The non-linear gradient was able to separate compounds of a wide polarity range as concluded after trying many linear and non-linear gradients. This selection was made considering the important factors of chromatography such as peak separation efficiency of the column, peak shape, total run time of gradient etc. The initial linear and non-linear gradients applied on columns 1, 2 and 3 are given in Supplementary Table S2. All the chromatograms for the respective gradients are given in Supplementary Figures S1–S3. The order of elution of individual standards using the final gradients on the three columns is presented in Supplementary Table S1.

To obtain better peak sensitivity, ELSD parameters such as drift tube temperature, nebulizing gas pressure and gain were optimized. At constant values of temperature, 80 °C and nebulizing gas pressure of 50 ± 3, different values of gain were used, and peak intensities were measured. It was noted that the gain value of 10 has much better peak intensities. By using the highest value of gain, 12, the noise level was also increased to a considerable level in addition to the peak intensities, which was undesirable. At lower gain values such as 5, low peak intensities were obtained. Using the optimized value of gain and nebulizing gas pressure value of 50 ± 3, the drift tube temperature varied from 60 °C at the start and increased by ten units after each injection (Table 3). At the highest temperature used, most of the peak intensities were lowered, probably due to heat degradation. The optimum results were obtained at a temperature of 60 °C. The effect of nebulizing gas pressure was then tested, starting from 45 ± 3 and increasing five units after each run. The results showed that increasing gas pressure promotes peak intensities and suppresses the noise level. Table 3 and Supplementary Figure S4 show the parameter optimization conditions and resulting profiles, respectively.

We tested a short column, i.e., column 1, to obtain faster run times. However, the resolution was not good enough to separate the triterpenes. Therefore, column 2 was used where the resulting chromatogram was better in terms of flavonoids peak resolution; however, no significant improvement of the triterpene’s separation was observed. Column 3 had smallest particle size and largest length as compared to the first two columns. This column resulted in the separation of all the standards, including the isomeric compounds, except one.

In addition to HPLC-ELSD analysis, the optimized chromatographic method was investigated using high-performance liquid chromatography with diode array detector (HPLC-DAD) as well. The wavelengths were selected to cover λmax values of all the flavonoids, sterols, and triterpenes in the current study. The best chromatogram obtained was that of 210 nm, in which the flavonoids and triterpenes exhibited varied peak intensities and shapes (Supplementary Figure S5). The peak shapes of triterpenes were not as good as those obtained using HPLC-ELSD.

### 2.2. Method Validation

Good linearity was observed with R2 values greater than 0.996 for every standard in the pool. The limit of detection (LOD) values ranged from 7.76 to 38.30 µg/mL and the limit of quantification (LOQ) ranged from 23.52 to 116.06 µg/mL (Table 4).

Precision was estimated by performing intra-day injections for repeatability and inter-day injections in three consecutive days. Four concentration levels of 870, 620, 400, and 200 µg/mL, were used in triplicate and expressed as percent relative standard deviation (% RSD). Most of the values obtained for precision and percent error were below 10, which shows good precision and trueness (Supplementary Table S4).

### 2.3. LC-ESI-MS/MS Analysis

All standards except compounds **10**, **11**, and **12** showed peaks at the expected values of retention times in positive ionization mode. However, in the negative ionization mode, betulin (**9**), a common triterpene, was missing. The absence of the compounds, both in positive and negative ionization modes, can be explained by the incompatibility of the ionization technique electrospray ionization mass spectrometry (ESI-MS) used with the nature of the highly non-polar compounds. Among the isomeric compounds (i.e., **6**, **7**, and **8**), **6** was differentiated based on its retention time, while the other two compounds were differentiated based on the mass to charge ratio (*m*/*z*) values of their fragments in the positive ionization mode. The ppm error was below 1, which showed the trueness of the data (Supplementary Table S5).

### 2.4. Comparison with Reported Methods

A thorough literature survey was carried out to find a chromatographic method that addresses the separation of most common plant secondary metabolites of different natures together in a single run, but nothing was found. The available chromatographic methods dealt mostly with compounds of a particular class. The equipment utilized in reported methods were also compared, and it was found that most studies had utilized very expensive techniques such as LC-MS. As the run times were assessed, it was found that some of the methods demonstrated short time intervals, especially those involving flavonoids, but those dealing with triterpenes and sterols were mainly long (Table 5).

As we compare the methods, we clearly see that none of the methods deals with all of the 12 compounds together, whereas the current method incorporates them all.

### 2.5. Analysis of Plant Extracts

Fourteen plant samples, Shf-1 to Shf-14, were injected in triplicate and the compounds under study were putatively identified based on the retention times obtained for standard compounds. Pool-1, which contained all the twelve standards, was injected in triplicate and the average retention times of individual standard peaks were noted. These retention time values of standards were used to identify peaks of interest in the extracts chromatograms. Furthermore, those peaks which were within the quantitative range were used to calculate concentrations (Table 6).

The plant samples extracts of ten different species were dissolved in different solvents such as methanol, ethyl acetate and pet ether. Flavonoids were found to be the most abundant secondary metabolites among the three classes. Quercetin (**3**), apigenin (**4**), and kaempferol (**5**), three known flavonoids, were detected in most of the plant samples. From triterpenoids and sterols classes, betulin (**9**), lupeol (**10**) and stigmasterol (**11**) were seen evidently. Betulin (**9**), was the most dominant particularly in the ethyl acetate extract of *Olea europea* leaves (Shf-9) i.e., 581.8 µg/mL, while kaempferol (**5**) showed its lowest detection level in the methanolic extract of *Polyalthia longifolia* var. pendula (P) root bark (Shf-6) as quantified to 53.0 µg/mL. The analysis of ethyl acetate extract of *Caesalpinia pulcherrima* (Shf-2), methanolic extract of dried leaves of *Polyalthia longifolia* (Shf-11) var. pendula (P), and methanolic extract of *Melia azedarach* (Shf-14) resulted in the identification of a total of nine compounds. The number of quantifiable compounds was five, which were identified in samples Shf-2, 9 and 11.

## 3. Materials and Methods

### 3.1. Chemicals and Standard Solutions

HPLC-grade acetonitrile and methanol were purchased from Merck (Kenilworth, NJ, USA). Ultra-pure water was obtained using Direct 16 Milli-Q purification system (Milli-pore Co., Bedford, MA, USA). Formic acid was purchased from Fluka (Seelze, Germany). Chemical standards of rutin (**1**), taxifolin (**2**), quercetin (**3**), apigenin (**4**), kaempferol (**5**), betulinic acid (**6**), oleanolic acid (**7**), betulin (**9**), lupeol (**10**), stigmasterol (**11**), β-sitosterol (**12**) and ursolic acid (**8**) were purchased from Sigma-Aldrich Chemical Co. (St. Louis, MO, USA).

### 3.2. Plant Extract Preparation

Crude extracts of different parts of ten plant species were previously prepared [34,35,36,37,38,39,40]. 10 mg of each plant extract was dissolved in 1 mL methanol prior to sonication and vigorous mixing using a vortex mixer was carried out. The solutions were then centrifuged at 10,000× *g* RPM for 15 min followed by filtration using polytetrafluoroethylene (PTFE) syringe filters (Membrane solutions) of pore size 0.22 µm. These samples were stored at −20 °C in the freezer for further use.

### 3.3. Stock and Standard Solutions

The 12 standard stock solutions were made by weighing 1 mg of each standard followed by dissolving it in 1 mL of methanol. A stock solution of the standard mix, Pool-1, was made for HPLC analysis by mixing the 12 standards to a final concentration of 1 mg/mL in methanol for each standard in the pool (1 mL each). Dissolving the residue in 1 mL of methanol was secondary to the mixing and evaporation of the solvent using a concentrator. The calibration curves of each standard in Pool-1 were obtained by using seven calibrators of 50, 100, 200, 300, 500, 750, 1000 µg/mL, prepared by the serial dilution method. The concentration levels made for QC samples (standards) were 870, 620, 400, and 200 µg/mL.

### 3.4. HPLC Analysis

The chromatographic analyses were performed using HPLC and evaporative light scattering detector (ELSD), both manufactured by Agilent Technologies (Santa Clara, CA, USA) 1200 series. Agilent (1200) ChemStation Rev. B.03.02 software was used for processing the data. The columns used for method optimization were Poroshell 120 EC-C18, Agilent Technologies (Santa Clara, CA, USA) with the dimensions of 3.0 × 50 mm and a pore size of 2.7 µm (column 1), ZORBAX Eclipse XDB-Phenyl, Agilent Technologies (Santa Clara, CA, USA) with the dimensions 4.6 × 75 mm and pore size 3.5 µm (column 2) and EC, NUCLEODUR C18 Gravity, Macherey-Nagel (Düren, Germany) with the dimensions 3 × 100 mm and pore size 1.8 µm (column 3). The column temperature was set at 30 °C and was kept constant throughout the analysis. Solvent A was ultra-pure water plus 0.1% formic acid and Solvent B was HPLC grade acetonitrile with 0.1% formic acid. The same mobile phase was used in all HPLC analyses. The optimized flow rate for column 1 was 1 mL/min, for column 2, 0.5 mL/min, and for column 3, 0.6 mL/min for the first 17 minutes then 1.3 mL/min for the next eight minutes and then 0.6 mL/min in the last four minutes. The injection volume was 1 µL for analysis of individual standards and plant samples. 1 µL of Pool-1 was used to obtain calibration curves and QC data. Using column 3, injection of Pool-1 was also made on HPLC system Agilent Technologies 1260 infinity which was coupled with DAD under the optimized chromatographic conditions. The wavelengths used were 210, 256, 270, 290, 330, 350, 370 nm.

### 3.5. HPLC-ESI-MS/MS Analysis

HPLC-ESI-MS/MS was performed employing gradient 7 on column 3 using the same mobile phase flow rate as in HPLC-ELSD. The instrument consisted of Bruker maXis IITM high resolution quadrupole time of flight (HR-QTOF) mass spectrometer (Bremen, Germany) coupled to Dionex UltiMateTM 3000 series HPLC system (Thermo Fisher Scientific, Waltham, MA, USA) fitted with a binary RS pump, an auto-sampler and column thermostat. The ion source used was ESI (electrospray ionization). MS and MS/MS spectra were obtained using both positive and negative modes. The ion source parameters were set as follows (parameters for negative mode next to positive mode parameters): capillary voltage at 4500 V (−3500 V), end plate offset at 500 V, drying gas temperature at 220 °C, drying gas at 8.0 L/min and nebulizer gas 45.0 psi. The mass range was *m*/*z* 100–2000 at the scan speed of 5 Hz for MS, while it was 12 Hz for MS/MS spectra.

Studies using liquid chromatography coupled with mass spectrometer was carried out using both positive and negative ionization modes in combination with electrospray ionization as ion source. A 0.3 min calibration segment was set before each LC-ESI-MS/MS run to achieve maximum trueness in data. During this time period, sodium formate was injected using the flow rate 3 µL/min. The solution of sodium formate was prepared as 10 mM in water: 2 propanol in the ratio of 1:1. The values obtained for *m*/*z* clusters of sodium formate were compared with those of the data obtained for calibration purpose. Calibration was carried out at high-precision calibration (HPC) mode. Various parameters such as the accurate mass, fragmentation pattern, and mSigma values were screened for each compound. The data analysis was performed using Bruker Compass DataAnalysis (ver. 4.4 SR1, 64-bit) and Bruker Compass TargetAnalysis (ver.1.3). Before analyzing the data, the spectral background subtraction algorithm built-in in DataAnalysis 4.4 was used for noise removal.

### 3.6. Method Validation

The optimized method was tested for validity by performing linearity, reproducibility, limit of detection and quantification, precision and trueness studies. Seven different concentration levels of standard (Pool-1) were made in the range of 50–1000 µL using appropriate dilutions of the stock solution. Calibration curves were obtained by plotting area values on the y-axis and concentration values on the x-axis. LOD and LOQ were calculated using the relations LOD = 3 σ/m, LOQ = 10 σ/m where σ represents standard deviation and m is the slope of the calibration curve. Precision was estimated by performing intra-day and inter-day injections in three days using triplicates and expressed as RSD. The relations, Precision (%RSD) = 100 × Standard deviation/Co and trueness (%Error) = 100 × (Ct − Co) /Ct, were used to calculate precision and trueness, respectively, where Co is the observed concentration, and Ct is the calculated one.

## 4. Conclusions

In the current study, an HPLC-ELSD method was developed and validated for the rapid identification of the twelve most common secondary metabolites of plant origin belonging to the classes of flavonoids, triterpenes and sterols. This high-throughput method can be of great potential for the future isolation of natural products from herbs employing the preliminary rapid screening. By identifying the most common secondary metabolites at the early stage, a researcher can save time by eliminating the reported compounds and avoid re-purification and re-isolation. Hence, there is a higher chance to selectively identify and work on the compounds of interest for further investigations and future implications.

## Figures and Tables

**Table 1:** Stationary phase optimization.

S. No.	Manufacturer	Column	Dimensions	Flow Rate	Temperature (°C)	Gradient Used	Well Resolved Peaks
				(mL/min), Time (min)		(Gradient No.)	
1	Agilent	Poroshell 120 EC-C18	3 × 50 mm, 2.7 µm	1	30	(4) 10% B, 0–1 min; 10–98% B, 1–5 min; 98% B, 5–7.5 min; 98–10% B, 7.5–9 min; 10% B, 9–10 min.	4
2	Agilent	ZORBAX Eclipse	4.6 × 75 mm, 3.5 µm	0.5	30	(4) 20% B, 0–2 min; 20–70% B, 2–15 min; 70–80% B, 15–20 min; 80–85% B, 20–21 min; 85–98% B, 21–27 min; 98–20% B, 27–28 min; 20% B, 28–30 min.	6
		XDB-Phenyl					
3	Macherey-Nagel	EC, NUCLEODUR C18 Gravity	100 × 3 mm, 1.8 µm	0.6, 0–17	30	(7) 20% B, 0–2 min; 20–45% B, 2–10 min; 45–85% B, 10–11 min; 85–88% B, 11–17 min; 88–100% B, 17–18 min; 100% B, 18–25 min; 100–20% B, 25–27 min; 20%B, 27–29 min.	11
				1.3, 17–25			
				0.6, 25–29			

**Table 2:** Chromatographic features on the optimized gradient method.

Compound	Rt ^a^ (Min)	Width (Min)	Rs ^b^
Rutin	2.33	0.13	9.11
Taxifolin	3.73	0.17	9.11
Quercetin	8.10	0.13	11.47
Apigenin	9.62	0.13	1.63
Kaempferol	9.91	0.16	0.17
Betulinic acid	16.03	0.15	2.62
Ursolic acid + Oleanolic acid	16.46	0.17	1.37
Betulin	16.74	0.23	1.37
Lupeol	22.55	0.11	1.68
Stigmasterol	24.50	0.15	6.10
β-sitosterol	25.44	0.16	6.10

^a^ retention time. ^b^ peak resolution.

**Table 3:** ELSD optimization.

ELSD Parameters	Optimization Parameters Condition			
	a	b	c	d
Nebulizer gas pressure (psi)	50 ± 3	50 ± 3	45 ± 3, 55 ± 3	55 ± 3
Photomultiplier gain	1, 3, 5, 7, 9, 10, 12	10	10	10
Drift tube temperature (°C)	80	60, 70, 90	60	60

a: gain optimization at constant temperature and nebulizing gas pressure. b: drift tube temperature optimization at constant pressure and optimized gain value. c: nebulizing gas pressure optimization at selected temperature and gain values. d: final optimized values.

**Table 4:** Calibration values (R^2^, LOD and LOQ).

S. No	Compound Name	Linear Calibration Range	Regression Equation	R^2^	LOD	LOQ
		(µg/mL)			(µg/mL)	(µg/mL)
1	Rutin	100–1000	y = 1.837x − 0.1568	0.997	29.4	89.1
2	Taxifolin	100–1000	y = 1.8246x − 0.153	0.996	31.3	94.9
3	Quercetin	100–1000	y = 3.5603x − 0.4755	0.999	19.1	57.9
4	Apigenin	200–1000	y = 2.6904x − 0.3257	0.997	38.3	116.1
5	Kaempferol	50–1000	y = 4.909x − 0.2025	0.999	15.8	47.9
6	Betulinic acid	50–1000	y = 3.8292x − 0.4187	0.999	7.8	23.5
7	Oleanolic acid + Ursolic acid	100–1000	y = 9.6962x − 0.84	0.996	29.9	90.5
8	Betulin	100–1000	y = 5.5929x − 0.6313	0.999	15.3	46.3
9	Lupeol	100–1000	y = 3.6515x − 0.4517	0.999	21.5	65.0
10	Stigmasterol	100–1000	y = 2.2809x − 0.2659	0.999	25.6	77.5

**Table 5:** Reported HPLC results of flavonoids, sterols, and triterpenes separation, and a comparison with the current study.

HPLC Column	Mode of Elution	Mobile Phase	Compounds												Run Time	Technique	Ref.
			1	2	3	4	5	6	7	8	9	10	11	12			
Hypersil BDS C 18 (250 × 3 mm) 3 μm	Isocratic	6.5% H_2_O in acetonitrile	-	-	-	-	-	-	-	-	-	+	+	+	35 min	HPLC-UV	[13,17]
Acquity BEH C18 (100 mm × 2.1 mm, 1.7 μm)	Gradient	Ultra-pure water and methanol	+	-	+	+	+	-	-	-	-	-	-	-	13 min	HPLC-MS	[25,31]
Mixed-Mode WAX-1 (2.1 × 150 mm) 3 μm	Isocratic	Formate buffer solution in acetonitrile	-	-	-	-	-	+	+	+	+	+	-	-	7 min	MMLC-MS	[26,32]
Zorbax Eclipse PAH (150 mm × 4.60 mm, 3.50 μm)	Gradient 1	Ultra-pure water with acetic acid 0.05% and methanol with acetic acid 0.05%	-	-	-	-	-	-	+	+	-	-	-	-	24 min	LC-APCI-MS	[27,33]
Zorbax Eclipse XDB-C18 (150 mm × 4.60 mm, 5.00 μm)	Gradient 2	Ultra-pure water with 0.025% acetic acid and solvent B, acetonitrile with 5% acetone	+	-	+	+	-	-	-	-	-	-	-	-	8.5 min	LC-ESI-MS/MS	
EC, NUCLEODUR C18 Gravity (100 mm × 3 mm), 1.8 µm	Gradient	Ultra-pure water with 0.1% formic acid and ACN with 0.1% formic acid	+	+	+	+	+	+	±	±	+	+	+	+	29 min	HPLC-ELSD	Current study

(±) merged peak. (-) missing peak. (+) peak present.

**Table 6:** Analysis of plant extracts.

S. No	Extract Code	Plant Source (Solvent)	Rutin	Taxifolin	Quercetin	Apigenin	Kaempferol	Betulinic Acid	Oleanolic Acid + Ursolic Acid	Betulin	Lupeol	Stigmasterol
1	Shf-1	*Caesalpinia pulcherrima*, flowers (ethyl acetate)	485.97	+	234.56	+	284.01	-	-	227.33	+	+
2	Shf-2	*Caesalpinia pulcherrima* fresh pods (ethyl acetate)	212.45	137.24	315.07	+	304.01	+	-	112.76	+	+
3	Shf-3	*Citrus lemon* seeds cover (ethyl acetate)	146.71	-	+	+	568.31	-	-	-	+	+
4	Shf-4	*Opuntia dellenii* cladodes (ethyl acetate)	+	+	-	435.70	374.07	-	-	212.24	-	-
5	Shf-5	*Bauhinia variegata* pod cover, (ethyl acetate)	482.39	+	237.84	468.80	+	-	+	265.92	-	+
6	Shf-6	*Polyalthia longifolia* var. pendula (P) root bark, (methanol)	+	-	579.51	+	53.06	-	-	+	+	413.35
7	Shf-7	*Bombax ceiba*, wood (methanol)	-	+	+	475.00	+	+	+	564.81	-	+
8	Shf-8	*Phlox drummondii* aerial part (Methanol:H_2_O)	-	+	+	+	+	-	-	-	+	-
9	Shf-9	*Olea europea* leaves, (ethyl acetate)	197.17	185.71	94.50	+	483.12	-	-	581.83	+	+
10	Shf-10	*Caesalpinia pulcherrima* flowers, (pet ether)	-	-	+	+	+	+	+	+	110.01	+
11	Shf-11	*Polyalthia longifolia* var. pendula (P) dried leaves, (methanol)	+	399.64	+	185.58	207.84	-	506.22	+	+	362.03
12	Shf-12	*Tagetes patula* flowers, capitulam (Pet ether)	+	-	-	399.88	-	-	-	466.82	+	-
13	Shf-13	*Bombax ceiba* stem bark (pet ether)	-	-	-	-	+	-	156.67	490.67	+	-
14	Shf-14	*Melia azedarach* flowers (Methanol)	223.89	+	+	+	496.56	-	+	378.49	+	+

Concentrations are given in µg/mL. (-) compound not detected. (+) compound is detected but below LOQ.

## Data Availability

The data presented in this study are available on request from the corresponding author. The data are not publicly available due to confidentiality.

## Supplementary Materials

The following are available online at https://www.mdpi.com/article/10.3390/metabo11080489/s1, Figure S1: Chromatograms, a–d obtained using column 1 with gradients 1–4 respectively, Figure S2: Chromatograms, a–d obtained using column 2 with gradients 1–4 respectively, Figure S3: Chromatograms, a–d obtained using column 2 with gradients 1–4 respectively, Figure S4: Effect of variation of temperature at constant pressure and gain values; a—60 °C, b—70 °C, c—80 °C, d—90 °C, Figure S5: HPLC-DAD profile obtained for Pool-1 using EC, NUCLEODUR C18 Gravity (100 × 3), 1.8 um, Table S1: Optimization of chromatographic features on different gradients, Table S2: Gradients tested on columns, 1, 2, and 3, prior to obtaining the final gradients, Table S3: Retention times of standards using three different columns, Table S4: %Accuracy and %RSD of compounds; Table S5: Data of compounds detected in Pool-1 (positive and negative ionization modes).
