# Supplementary material for: Rapid Identification of Common Secondary Metabolites of Medicinal Herbs Using High-Performance Liquid Chromatography with Evaporative Light Scattering Detector in Extracts
Source: Metabolites. 2021 Jul 28;11(8):489. doi: 10.3390/metabo11080489 (PMC8398965; doi:10.3390/metabo11080489)
Supplement: Supplementary file 1 [file metabolites-11-00489-s001.zip › metabolites-1303422-SI.pdf]

Supplementary material

Rapid Identification of Common Secondary Metabolites of Medicinal Herbs Using High-Performance Liquid Chromatography with Evaporative Light Scattering Detector in Extracts

Kiran Ali[a], Arslan Ali[b], Muhammad Noman Khan[a], Saeedur Rahman[a], Shaheen Faizi[a], Muhammad Shaiq Ali[a], Shaden A. M. Khalifa[c], Hesham R. El-Seedi[c,d,e*] and Syed Ghulam Musharraf[a,b,f,*]

[a] *H.E.J. Research Institute of Chemistry, International Center for Chemical and Biological Sciences, University of Karachi, Karachi-75270, Pakistan.*

[b] *Dr. Panjwani Center for Molecular Medicine and Drug Research, International Center for Chemical and Biological Sciences, University of Karachi, Karachi-75270, Pakistan.*

[c] *Department of Molecular Biosciences, Stockholm University, the Wenner-Gren Institute, SE-106 91 Stockholm, Sweden.*

[d] *Department of Chemistry, Faculty of Science, Menoufia University, 32512 Shebin El-Kom, Egypt*

[e] *International Research Center for Food Nutrition and Safety, Jiangsu University, Zhenjiang 212013, China.*

[f] *T.C.M. Hospital of Southwest Medical University, Luzhou, Sichuan, China.*

Correspondence:

Prof. Dr. Syed Ghulam Musharraf

H.E.J. Research Institute of Chemistry, International Center for Chemical and Biological Sciences, University of Karachi, Karachi-75270, Pakistan.

Tel.: +92 213 4824924-5; 4819010; fax: + 92 213 4819018-9.

E-mail address: musharraf1977@yahoo.com

Prof. Dr. Hesham R. El-Seedi

Pharmacognosy Group, Department of Pharmaceutical Biosciences, BMC, Uppsala University, SE-751 23 Uppsala, Sweden.

Tel.: +46 73 5668234

E-mail address: hesham.el-seedi@farmbio.uu.se

Supplementary Figure Legends

Supplementary Fig. S1. Chromatograms, a - d obtained using column 1 with gradients 1- 4 respectively.

Supplementary Fig. S2. Chromatograms, a - d obtained using column 2 with gradients 1- 4 respectively.

Supplementary Fig. S3. Chromatograms, a - d obtained using column 2 with gradients 1- 4 respectively.

Supplementary Fig.S4. Effect of variation of temperature at constant pressure and gain values; a-60℃, b-70℃, c-80℃, d-90℃

Supplementary Fig. S5. HPLC-DAD profile obtained for Pool-1 using EC, NUCLEODUR C18 Gravity (100 x 3), 1.8 um.

Supplementary Table Legends

Supplementary Table S1. Optimization of chromatographic features on different gradients.

Supplementary Table S2. Gradients tested on columns, 1, 2, and 3, prior to obtaining the final gradients.

Supplementary Table S3. Retention times of standards using three different columns.

Supplementary Table S4. %Accuracy and %RSD of compounds.

Supplementary Table S5. Data of compounds detected in Pool-1 (positive and negative ionization modes).

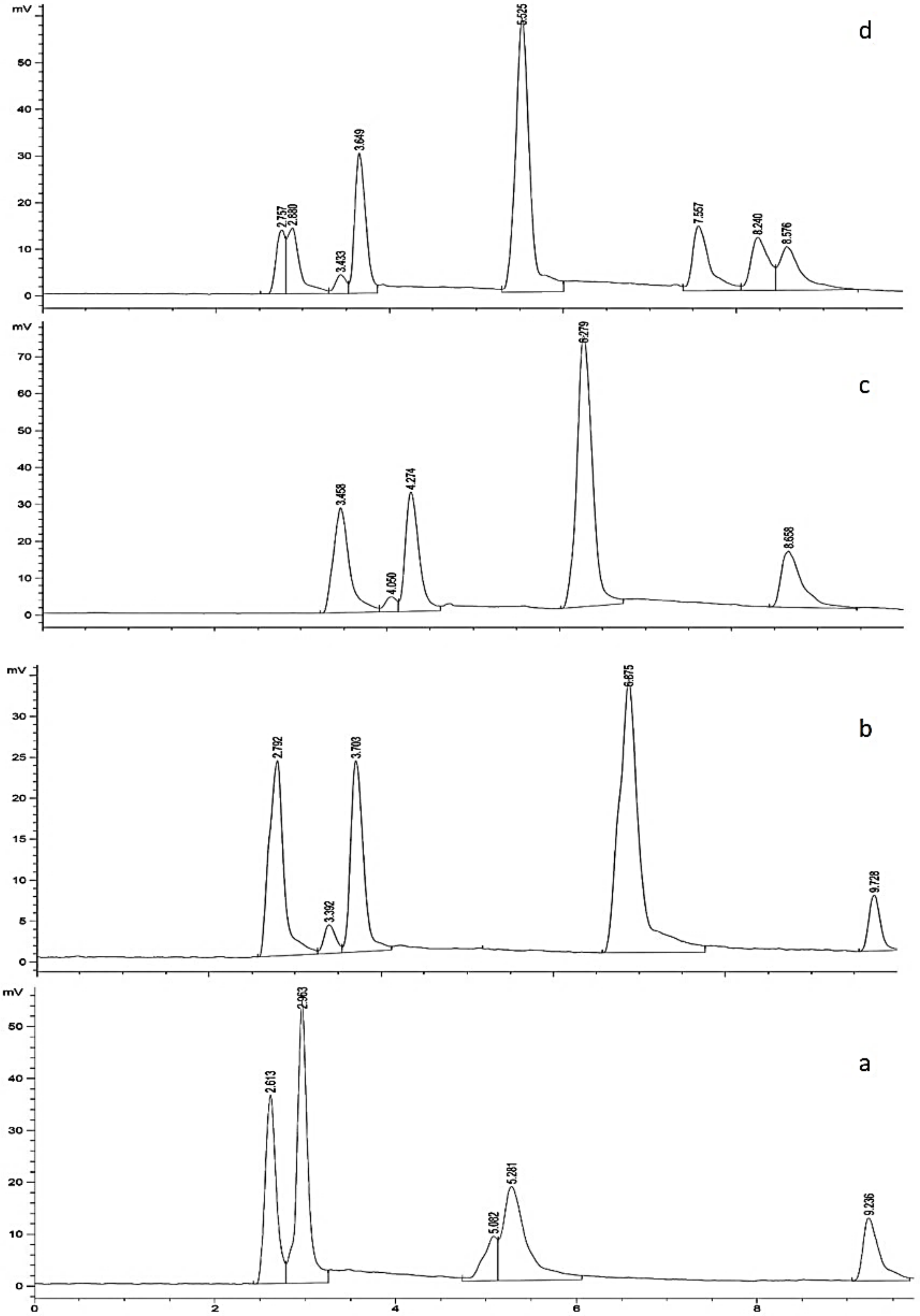

Supplementary Fig. S1. Chromatograms, a - d obtained using column 1 with gradients 1- 4 respectively.

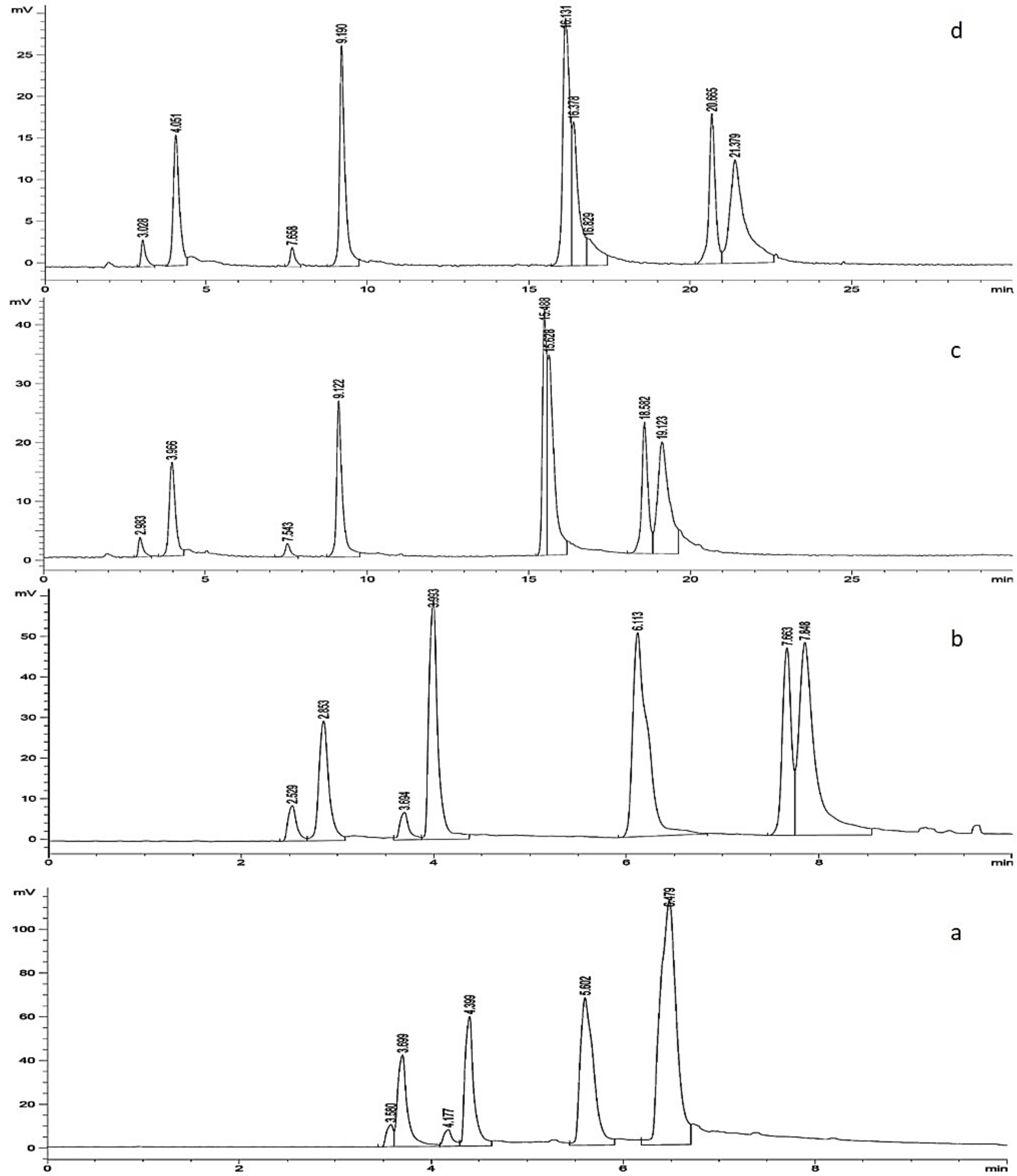

Supplementary Fig. S2. Chromatograms, a - d obtained using column 2 with gradients 1- 4 respectively.

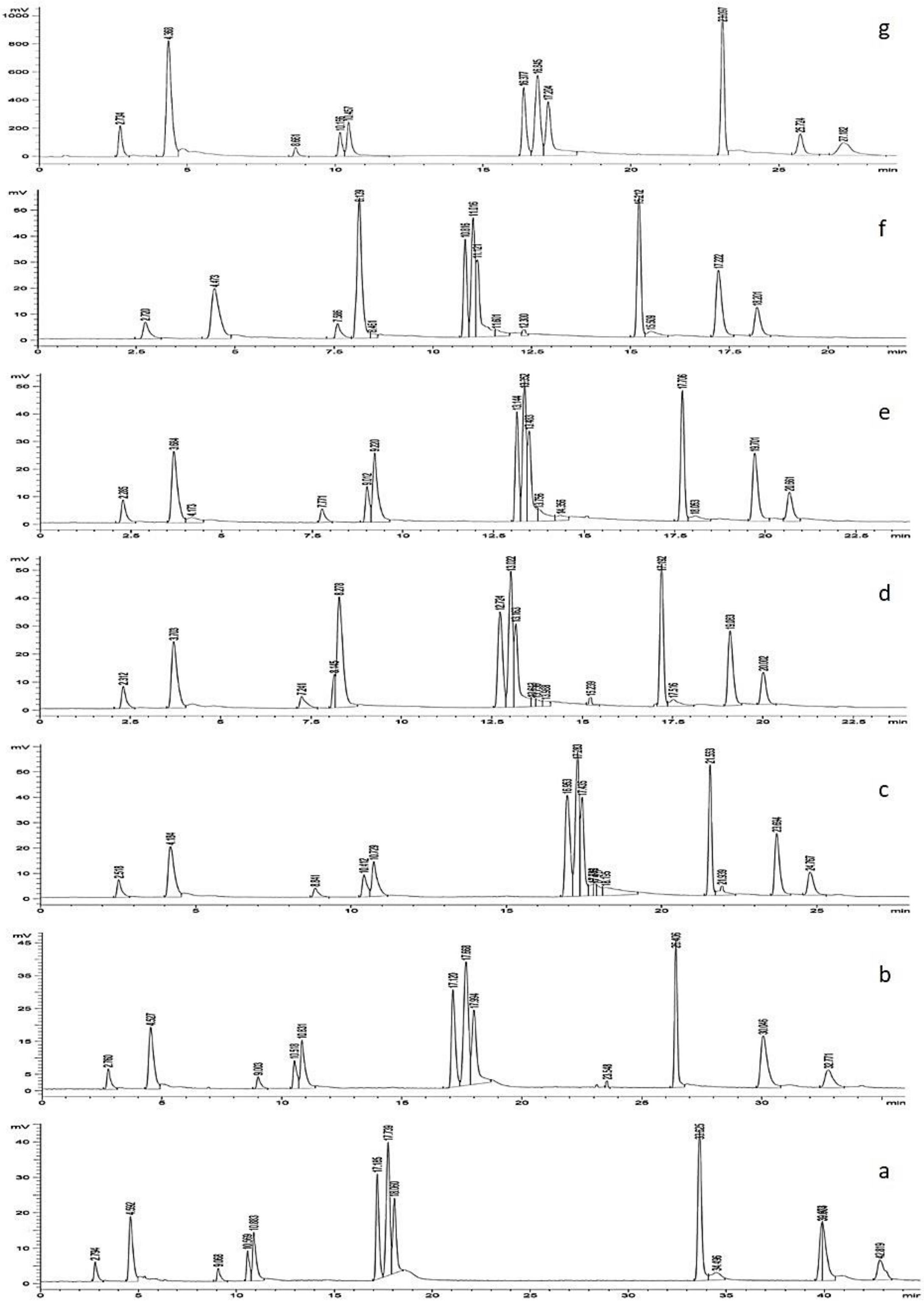

Supplementary Fig. S3. Chromatograms, a - g obtained using column 3 with gradients 1- 7 respectively.

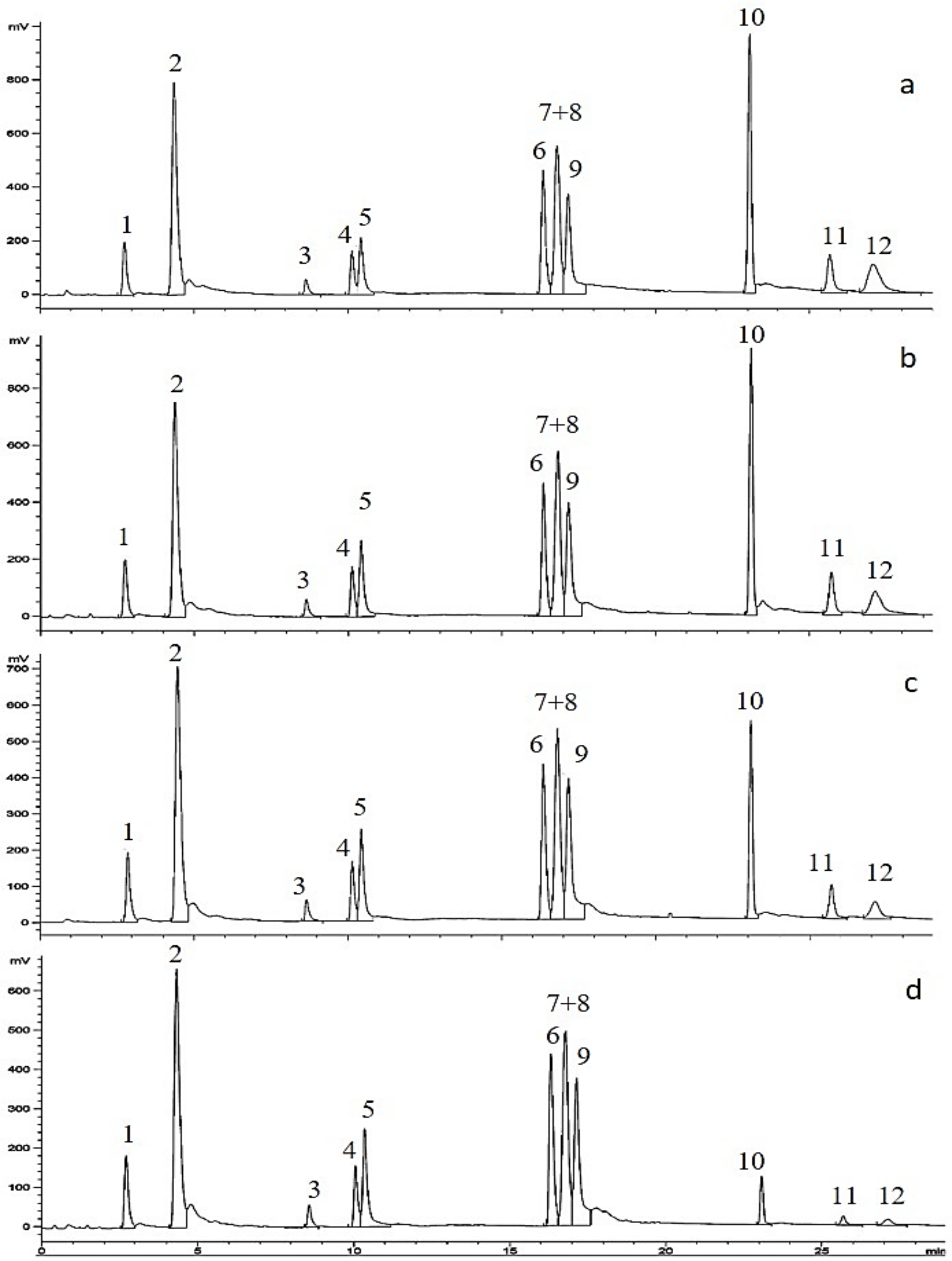

Supplementary Fig.S4. Effect of variation of temperature at constant pressure and gain values; a-60℃, b-70℃, c-80℃, d-90℃

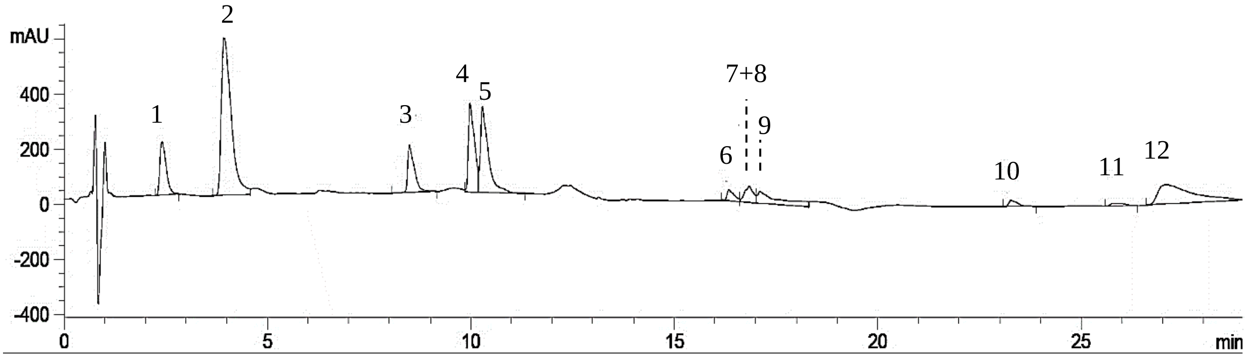

Supplementary Fig. S5. HPLC-DAD profile obtained for Pool-1 at 210 nm, using EC, NUCLEODUR C18 Gravity (100 x 3), 1.8 um.

Supplementary Table S1. Optimization of chromatographic features on different gradients.

Compound	Gradient method	Rt	Width	Rs
Quercetin	1	9.068	0.1494	10.090
Quercetin	2	9.003	0.1635	9.834
Quercetin	3	8.841	0.1573	10.271
Quercetin	4	7.241	0.1633	7.349
Quercetin	5	7.771	0.1274	10.303
Quercetin	6	7.586	0.1328	4.247
Quercetin	7	8.102	0.1312	11.470
Rutin	1	2.794	0.1632	9.78
Rutin	2	2.760	0.1614	9.83
Rutin	3	2.518	0.1441	9.82
Rutin	4	2.312	0.1306	9.30
Rutin	5	2.285	0.1344	9.27
Rutin	6	2.720	0.1593	9.54
Rutin	7	2.325	0.1337	9.11
Taxifolin	1	4.592	0.2042	9.78
Taxifolin	2	4.527	0.1981	9.83
Taxifolin	3	4.184	0.1950	9.82
Taxifolin	4	3.703	0.1684	9.30
Taxifolin	5	3.684	0.1673	9.27
Taxifolin	6	4.473	0.2082	9.54
Taxifolin	7	3.730	0.1745	9.11
Apigenin	1	10.569	0.1481	1.89
Apigenin	2	10.518	0.1446	1.93
Apigenin	3	10.412	0.1486	1.98
Apigenin	4	8.145	0.0827	1.06
Apigenin	5	9.012	0.1135	1.626

Apigenin	6	8.139	0.1276	0
Apigenin	7	9.615	0.1326	0.169
Kaempferol	1	10.883	0.1839	1.89
Kaempferol	2	10.831	0.1794	1.93
Kaempferol	3	10.729	0.1700	1.98
Kaempferol	4	8.278	0.1662	1.06
Kaempferol	5	9.220	0.1423	1.626
Kaempferol	6	8.139	0.1276	0
Kaempferol	7	9.906	0.1572	0.169
Betulinic acid	1	17.185	0.1640	3.07
Betulinic acid	2	17.120	0.1682	2.89
Betulinic acid	3	16.953	0.1691	2.19
Betulinic acid	4	12.724	0.1476	2.25
Betulinic acid	5	13.144	0.1027	1.94
Betulinic acid	6	10.816	0.0963	1.98
Betulinic acid	7	16.032	0.1541	2.62
Ursolic acid + Oleanolic acid	1	17.739	0.1965	1.69
Ursolic acid + Oleanolic acid	2	17.668	0.2110	1.54
Ursolic acid + Oleanolic acid	3	17.283	0.1320	1.22
Ursolic acid + Oleanolic acid	4	13.022	0.1171	1.13
Ursolic acid + Oleanolic acid	5	13.352	0.1111	1.18
Ursolic acid + Oleanolic acid	6	11.016	0.1053	0.97
Ursolic acid + Oleanolic acid	7	16.462	0.1729	1.37
Betulin	1	18.060	0.1832	1.69
Betulin	2	17.994	0.2103	1.54
Betulin	3	17.435	0.1161	1.22
Betulin	4	13.163	0.1309	1.13
Betulin	5	13.483	0.1107	1.18

Betulin	6	11.121	0.1111	0.97
Betulin	7	16.739	0.2304	1.37
Lupeol	1	33.625	0.1932	2.81
Lupeol	2	26.406	0.1295	17.42
Lupeol	3	21.553	0.1056	2.66
Lupeol	4	17.192	0.1060	1.73
Lupeol	5	17.706	0.1093	1.75
Lupeol	6	15.212	0.1035	1.67
Lupeol	7	22.552	0.1056	1.68
Stigmasterol	1	39.904	0.1871	11.82
Stigmasterol	2	30.046	0.2882	9.26
Stigmasterol	3	23.694	0.1678	5.81
Stigmasterol	4	19.083	0.1523	5.97
Stigmasterol	5	19.701	0.1657	5.70
Stigmasterol	6	17.222	0.1545	6.12
Stigmasterol	7	24.497	0.1517	6.10
Betasitosterol	1	42.819	0.3061	11.82
Betasitosterol	2	32.771	0.2998	9.26
Betasitosterol	3	24.767	0.2029	5.81
Betasitosterol	4	20.002	0.1554	5.97
Betasitosterol	5	20.661	0.1710	5.70
Betasitosterol	6	18.201	0.1651	6.12
Betasitosterol	7	25.441	0.1577	6.10

Supplementary Table S2. Gradients tested on columns, 1, 2, and 3, prior to obtaining the final gradients.

Columns	Gradient 1			Gradient 2			Gradient 3			Gradient 4			Gradient 5			Gradient 6		
	Time	% B	Flow Rate (mL/min)	Time	% B	Flow Rate (mL/min)	Time	% B	Flow Rate (mL/min)	Time	% B	Flow Rate (mL/min)	Time	% B	Flow Rate (mL/min)	Time	% B	Flow Rate (mL/min)
column 1	1	10	1	1	10	1	1	10	0.7									
	2	65	1	1.5	25	1	5	98	0.7									
	4	65	1	7.5	98	1	7.5	98	0.7									
	4.5	80	1	8.5	98	1	9	10	0.7									
	6.5	80	1	9	10	1	10	10	0.7									
	7	98	1	10	10	1												
	8.5	98	1															
	9	10	1															
	10	10	1															
column 2	0	10	1	0	20	1	0	20	0.5									
	1	10	1	1	20	1	2	20	0.5									
	5	98	1	2	40	1	10	50	0.5									
	7.5	98	1	7.5	98	1	10.5	50	0.5									
	9	10	1	8.5	98	1	12	70	0.5									
	10	10	1	9	20	1	25	98	0.5									
				10	20	1	26	98	0.5									
							28	20	0.5									
							30	20	0.5									
column 3	0	20	0.5	0	20	0.5	0	20	0.5	0	20	0.6	0	20	0.6	0	20	0.5
	2	20	0.5	2	20	0.5	2	20	0.5	2	20	0.6	2	20	0.6	2	20	0.5
	10	45	0.5	10	45	0.5	10	45	0.5	4	31	0.6	6	35	0.6	4	31	0.5
	11	85	0.5	11	85	0.5	12	87	0.5	4.5	39	0.6	7.5	45	0.6	4.5	39	0.5
	20	88	0.5	20	88	0.5	17	100	0.5	5.5	43	0.6	9	88	0.6	5.5	43	0.5
	22	91	0.5	21	100	1	17.5	100	1.3	6.5	85	0.4	12	89	0.6	7	85	0.5
	22.5	100	0.5	26	100	1	24	100	1.3	9	86.5	0.5	13.5	100	1.3	8	86.5	0.8
	40	100	0.5	31	100	0.5	26	20	0.5	11	88	0.5	20.5	100	1.3	10	88	0.8
	43	20	0.5	34	20	0.5	28	20	0.5	12.5	100	0.5	22	20	0.6	10.5	100	1.3
	45	20	0.5	36	20	0.5				13	100	1.3	24	20	0.6	18.5	100	1.3
										20.5	100	1.3				20.5	20	0.5
										22.5	20	0.5				22	20	0.5
										24	20	0.5						

Supplementary Table S3. Retention times of standards using three different columns.

HPLC column	Flow rate (mL/min)	Standard compounds											
		2	1	3	4	5	6	7	8	9	10	11	12
Poroshell 120 EC-C18 (3.0 x 50 mm) 2.7µm	1mL/min	2.76	2.93	3.46	3.64	3.70	5.48	5.58	5.61	5.53	7.59	8.28	8.65
ZORBAX Eclipse XDB-Phenyl (4.6 x 75mm) 3.5µm	0.5mL/min	4.17	3.13	7.78	9.26	9.30	16.30	16.20	16.21	16.49	20.80	21.39	21.56
EC, NUCLEODUR C18 Gravity (100 x 3), 1.8 µm	0.6mL/min To 1.3 mL/min	4.36	2.75	8.64	10.14	10.43	16.35	16.80	16.80	17.16	23.06	25.66	27.07

Supplementary Table S4. %Accuracy and %RSD of compounds.

Compound Name	Concentrations in µg/L	Intra-day			Inter-day		
		Found concentration in µg/mL	%RSD	%Accuracy	Found concentration in µg/mL	%RSD	%Accuracy
Rutin	200	185.35±0.01	2.86	92.67	191.46±0.00	0.74	95.73
	400	466.01±0.01	1.38	116.50	454.77±0.01	1.37	113.69
	620	726.61±0.04	5.94	117.19	694.12±0.02	2.74	111.96
	870	884.44±0.08	8.58	101.66	876.11±0.01	1.56	100.70
Taxifolin	200	140.98±0.00	1.73	70.5	156.60±0.01	7.75	78.30
	400	311.78±0.01	2.71	77.9	313.73±0.01	2.39	78.43
	620	494.84±0.03	5.32	79.8	513.37±0.01	2.27	82.80
	870	554.23±0.02	4.31	63.7	620.68±0.04	6.91	71.34
Quercetin	200	212.09±0.00	0.50	106.04	210.38±0.00	0.70	105.19
	400	469.77±0.01	1.91	117.44	462.57±0.00	0.53	115.64
	620	743.70±0.05	7.21	119.95	691.92±0.01	1.22	111.60
	870	909.98±0.06	6.23	104.59	877.88±0.01	0.90	100.91
Apigenin	200	215.74±0.00	2.28	107.86	210.89±0.00	0.40	105.45
	400	463.15±0.01	2.53	115.78	455.82±0.00	0.46	113.95
	620	725.22±0.05	7.29	116.97	682.26±0.01	1.54	110.04
	870	845.62±0.02	2.33	97.19	855.72±0.01	0.74	98.36
Kaempferol	200	163.83±0.01	4.10	81.91	170.40±0.00	1.99	85.20

	400	458.30±0.01	2.25	114.57	451.29±0.01	1.19	112.82
	620	722.72±0.01	1.95	116.56	697.09±0.01	1.72	112.43
	870	858.57±0.07	7.88	98.68	874.70±0.01	1.21	100.5
**Betulinic acid**	200	207.14±0.00	0.77	103.57	206.42±0.01	2.93	103.21
	400	489.35±0.02	3.13	122.33	476.55±0.00	0.77	119.14
	620	753.02±0.03	4.16	121.45	704.98±0.02	2.29	113.71
	870	902.88±0.04	4.36	103.77	866.14±0.01	1.62	99.56
**Oleanolic acid + Ursolic acid**	200	199.08±0.00	1.73	99.53	201.75±0.00	0.83	100.88
	400	520.83±0.02	3.68	130.20	502.17±0.00	0.38	125.54
	620	786.26±0.04	4.92	126.81	775.23±0.03	3.75	125.04
	870	942.50±0.01	0.97	108.33	884.88±0.04	4.79	101.71
**Betulin**	200	213.89±0.00	1.07	106.94	210.05±0.00	1.17	105.02
	400	513.00±0.02	3.40	128.25	500.84±0.01	1.72	100.84
	620	780.05±0.04	4.65	125.81	760.85±0.02	2.41	122.72
	870	931.76±0.06	6.26	107.09	912.80±0.04	4.41	104.9
**Lupeol**	200	203.87±0.00	1.06	101.93	200.35±0.00	0.54	100.18
	400	461.61±0.00	1.00	115.40	454.45±0.00	0.68	113.61
	620	716.61±0.01	0.88	115.58	691.09±0.02	3.59	111.47
	870	895.35±0.04	4.26	102.91	850.24±0.02	1.80	97.73
**Stigmasterol**	200	207.82±0.00	0.69	103.91	204.81±0.01	3.47	102.41
	400	464.49±0.01	1.35	116.12	455.42±0.00	0.85	113.85

	620	671.42±0.04	5.67	108.29	643.43±0.01	1.34	103.78
	870	658.36±0.00	0.42	75.67	688.68±0.04	5.98	79.16

Supplementary Table S5. LC-ESI-MS/MS data of compounds detected in Pool-1 (positive and negative ionization modes).

S. No.	Compound name	Formula	RT (min)	Ion Type	m/z measured (intensity)	m/z calculated	Error (ppm)	mSigma	MS/MS
1	Rutin	$C_{27}H_{30}O_{16}$	2.02	$[M+H]^+$<br><br>$[M-H]^-$	611.1604 ($3.41x10^5$)<br><br>609.1472 ($4.30x10^5$)	611.1607<br><br>609.1461	0.4<br><br>-1.8	1.7<br><br>1.4	303.0497, 229.0495, 305.0551 304.053<br><br>203.0121, 300.0281, 301.0348 301.0462, 609.1449, 610.1527
2	Taxifolin	$C_{15}H_{12}O_7$	3.2	$[M+H]^+$<br><br>$[M-H]^-$	305.0654 ($3.02x10^5$)<br><br>303.0516 ($6.51x10^5$)	305.066<br><br>303.051	0.5<br><br>-1.8	2.3<br><br>2.1	149.0234, 153.0184, 154.0225, 167.0341, 185.0603, 195.0287, 213.0551, 231.0655, 232.0684, 241.0493, 259.0604, 260.0645<br><br>241.0501, 151.0427, 153.0198 174.9533, 175.0397, 176.5807 177.0198, 178.9988, 199.0401 217.0499, 218.0535, 235.0622 259.059, 275.0567, 285.04 286.0419, 492.1735
3.	Quercetin	$C_{15}H_{10}O_7$	6.87	$[M+H]^+$<br><br>$[M-H]^-$	303.0498 ($1.46x10^5$)<br><br>301.0357 ($3.65x10^5$)	303.0499<br><br>301.0354	0.5<br><br>-1	3.5<br><br>1.5	137.0233, 153.0183, 154.0219, 155.0495, 161.0594, 163.0392, 165.0185, 173.0599, 179.0338, 183.0439, 187.0392, 201.0546, 202.0579, 211.0393, 228.0419, 229.0496, 230.0534, 239.0334, 245.0449, 247.061, 257.0445, 258.0484, 274.0475, 285.0398, 303.05, 304.0521<br><br>151.0041, 170.2741, 178.9988 186.9933, 257.0501, 271.0268 273.0415, 299.0215, 301.0351 301.0555, 302.0383, 302.0505
4.	Apigenin	$C_{15}H_{10}O_5$	8.35	$[M+H]^+$<br><br>$[M-H]^-$	271.06 ($6.12x10^5$)<br><br>269.0459 ($7.31x10^5$)	271.0601<br><br>269.0455	0.6<br><br>-1.3	1.7<br><br>15.8	145.0282,153.0182, 154.0213, 163.0386, 197.0592, 225.0545, 229.0498, 243.0648, 271.0599, 272.0635<br><br>149.0235, 269.0453, 270.0484 270.0644, 271.0531

5.	Kaempferol	$C_{15}H_{10}O_6$	8.62	$[M+H]^+$<br><br>$[M-H]^-$	287.055 ($3.95x10^5$)<br><br>285.0409 ($7.30x10^5$)	287.055<br><br>285.0405	0.2<br><br>-1.4	2.8<br><br>17.3	147.044, 153.0181, 154.0214 157.0647, 161.0593, 165.0181 171.0441, 184.0515, 185.0596 213.0544, 214.0583, 229.0498 231.0648, 241.049, 258.052 259.0588, 287.0548, 288.0585<br><br>240.854, 285.039, 286.0428 286.081, 287.045
6.	Betulinic acid	$C_{30}H_{48}O_3$	14.33	$[M+H]^+$<br><br>$[M-H]^-$	457.3676 ($4.03x10^3$)<br><br>455.3518 ($4x10^4$)	457.3676<br><br>455.351972	0<br><br>0.2	28.9<br><br>5.3	147.1178, 149.1312, 161.1314 163.1487, 173.1318, 175.1112 175.1476, 187.1107, 189.1628 203.1793, 337.2165<br><br>248.9604, 409.2434, 409.2614 410.2483, 455.3525, 456.3564 457.3593
7.	Oleanolic acid	$C_{30}H_{48}O_3$	14.71	$[M+H]^+$	457.3676 ($1.04x10^3$)	457.3657	0.1	16.5	149.1312, 308.27, 417.33
8.	Ursolic acid	$C_{30}H_{48}O_3$	14.71	$[M+H]^+$	457.3679 ($1.29x10^4$)	457.3676	0.1	16.5	149.1361, 161.1323, 191.1798
9.	Betulin	$C_{30}H_{50}O_2$	16.02	$[M+H]^+$<br><br>$[M-H]^-$	443.3883<br><br>($4.92x10^3$)	443.3884<br><br>441.372707	0<br><br>NF	9.4<br><br>NF	147.1163, 149.132, 161.1325 163.148, 177.1627, 189.1634 191.1776<br>NF
10.	Lupeol	$C_{30}H_{50}O$	NF	$[M+H]^+$<br>$[M-H]^-$	NF	427.393443<br>425.377793	NC	NC	NF
11.	Stigmasterol	$C_{29}H_{48}O$	NF	$[M+H]^+$<br>$[M-H]^-$	NF	413.377793<br>411.362143	NC	NC	NF

12.	Betasitosterol	$C_{29}H_{50}O$	NF	$[M+H]^+$<br>$[M-H]^-$	NF	415.393443<br>413.377793	1NC	NC	NF

NF not found

NC not calculated
